# Regulating Emotion in the Context of Interpersonal Decisions: The Role of Anticipated Pride and Regret

**DOI:** 10.3389/fpsyg.2012.00513

**Published:** 2012-11-19

**Authors:** Job van der Schalk, Martin Bruder, Antony Manstead

**Affiliations:** ^1^School of Psychology, Cardiff UniversityCardiff, UK; ^2^Department of Psychology, Zukunftskolleg, University of KonstanzKonstanz, Germany

**Keywords:** anticipated emotions, fairness, ultimatum bargaining game, pride, regret

## Abstract

Recent theories about the relation between emotion and behavior hold that social behavior is influenced not only by the experience of emotion, but also by the anticipation of emotion. We argue that anticipating future emotional states is an emotion regulation strategy when it leads to a change in behavior. In the current studies we examined how construal of a fair or an unfair situation in terms of positive or negative anticipated emotions influences the fairness of subsequent behavior. We used the Ultimatum Bargaining Game – an experimental game in which participants divide a resource between themselves and another person – as a social situation that offers the opportunity to engage in fair and unfair behavior. In Study 1 we used an autobiographical recall task to manipulate anticipated emotions. Although the task did not influence anticipated emotions directly, results showed that anticipated pride about fair behavior increased levels of fairness, whereas anticipated pride about unfair behavior decreased levels of fairness. Similarly, anticipated regret about fair behavior decreased levels of fairness, whereas anticipated regret about unfair behavior increased levels of fairness. In Study 2 we replicated this pattern of findings, and found that participants who thought about their anticipated emotions (pride or regret) in relation to unfair behavior behaved more fairly. We discuss these findings in relation to theories of emotion regulation and economic decision-making.

## Introduction

One way of regulating emotions is to anticipate how one’s actions will make one feel and to adjust one’s behavior accordingly. In terms of Gross and Thompson’s (2007) process model of emotion regulation, this form of regulation belongs to the category of “situation modification,” which refers to efforts to change a situation so as to modify its emotional impact. Anticipating how you would feel if you were to behave one way rather than another, and then deciding to act in the way that evokes desired emotions and avoids undesirable emotions is therefore an emotion regulation strategy. Although this way of regulating emotions applies to a wide variety of settings, both intrapersonal and interpersonal, we focus in the present research on interpersonal behavior, where the emotions concerned are ones relating to outcomes for the self vs. outcomes for another person. We focus on pride and regret relating to decisions to act fairly, in the sense of an equal distribution of resources between self and other, or unfairly, in the sense of retaining a larger proportion of the resource for oneself. We show that the extent to which one anticipates feeling proud or regretful about either course of action is related systematically to how one then decides to allocate resources between self and other.

Our theoretical reasoning derives from theory and research on anticipated emotions. In particular, we draw on the dual-process model proposed by Baumeister et al. (2007). These theorists distinguish between “automatic affect” and “conscious emotion.” The former is quick and can operate without awareness. The latter is slower, requiring more processing resources, and is by definition something of which the individual is aware. These two types of affective reaction are seen as having different relationships with behavior. As Baumeister et al. (2007, p.169) put it, “[E]motion may be rather too slow to guide behavior directly in a fast-changing situation, because time is required for the cognitive processing of the event to lead to physiological changes such as arousal, which in turn may activate motor responses. In contrast, automatic affect will arise almost instantaneously and therefore be available to steer behavior even at a moment’s notice.”

If conscious emotion is too slow to have a direct impact on behavior, what is its function? Baumeister et al. (2007) argue that its most important function is to establish the conditions for being able to anticipate future emotional reactions and thereby the capacity to modify one’s behavior so as to evoke desired emotions and avoid undesirable ones. This is achieved by stimulating conscious reflection about one’s past behavior and by leaving an “affective residue.” Acting in a way that is regretted and gives rise to guilt stimulates reflection about the action and tags the action with a negative affective residue. This is a resource that can be drawn on in future settings that involve similar features: “Emotion provides feedback about recent actions and, by implication, about the adequacy of the current if-then rules on which those actions were based. … Positive emotions generally validate the existing rules because those emotions signify that what the person did turned out well, and so the existing rules were presumably effective. … Negative emotions signal that one’s behavior was not successful, and hence they suggest that the if-then rules need to be revised” (Baumeister et al., 2007, p. 173).

Adults have all experienced past situations in which that have had to choose between acting in their own interests, regardless of others, or in the interests of others. Each course of action has advantages and disadvantages in the form of material and psychological outcomes. Depending on individual dispositions, these past experiences are ones that may have aroused pride (about having stood up for oneself, or about having acted fairly) or regret (about having been unfair, or about having ceded too much to the other). There is a large literature demonstrating that the anticipation of regret is a powerful motivator of strategic social decision-making (e.g., Zeelenberg, 1999). Although there is less research on the effects of anticipated pride in such situations, there is reason to believe that anticipated pride plays an important role in encouraging behavior that conforms to social standards (Tangney et al., 2007). On the basis of Baumeister et al.’s (2007) dual-process model, we argue that these past experiences of pride and regret gave rise to conscious reflection about how one acted (e.g., counterfactual thinking), and (in the case of regret) a revision of the if-then rules that guided the past action. When similar decisions have to be made in the future, people anticipate how they would feel if they were to act in accordance with these if-then rules. Anticipating these emotional consequences is likely to play a role in how people decide (see also Mellers et al., 1999; Loewenstein and Lerner, 2003).

Based on the above reasoning, we predicted that individuals who anticipate pride about acting fairly would be more likely to divide resources between themselves and another in a fair way, whereas those who anticipate regret about acting fairly would be less likely to do so. Similarly, we predicted that individuals who anticipate pride about acting unfairly would be less likely to divide resources between themselves and another in a fair way, whereas those who anticipate regret about acting unfairly would be more likely to do so. We conducted two online studies to investigate these hypotheses. In both studies participants played an economic game, the Ultimatum Bargaining Game (UBG; Güth et al., 1982), which was used as a measure of fairness of resource allocation. It could be argued that other moral emotions might also be relevant in social bargaining situations such as the UBG. These include self-conscious emotions such as shame and guilt, and anger-related responses such as moral outrage. Note, however, that we are interested in the interaction between the salience of social norms concerning fairness and unfairness and the anticipation of emotions associated with actually behaving in a fair or unfair manner. We therefore chose emotions that are applicable to both fair and unfair behavior. Pride and regret are psychologically plausible responses to both fair and unfair behavior, whereas emotions such as shame, guilt, or moral outrage, apply to unfair behavior but not to fair behavior.

There is a body of research on the role of emotion in the UBG, but that work has focused for the most part on the roles of anger, aggression, and reputation management on the part of responders in rejecting offers perceived to be unfair (e.g., Pillutla and Murnighan, 1996; Sanfey et al., 2003; Burnham, 2007). By contrast, our focus is on the role of emotions on the part of proposers, and how these emotions shape the offers they make. Recent work suggests that emotions do play a role in determining the offers made by proposers. For example, Martinez et al. (2011) found that proposers who were led to experience regret made higher offers than proposers in a neutral emotional state, whereas proposers who were led to experience disappointment made lower offers than their emotionally neutral counterparts.

More directly relevant to the current research is work reported by Nelissen et al. (2011, Study 1), in which they observed that proposers’ offers were influenced by the fear that they anticipated experiencing if their offers were rejected and the guilt they anticipated experiencing if their offers were thought to be inadequate. This provides initial evidence in support of the argument that proposers take the likely emotional consequences of their decisions into account when making offers. The explanation offered by Nelissen et al. (2011) for their findings was that anticipated fear and guilt reflect underlying concerns (concern for rejection, and concern for other player, respectively). This explanation is compatible with the present argument that anticipated emotion shapes the decision-making process by signaling to proposers how they would feel if they were to act in one way rather than another. This affective forecasting (Wilson and Gilbert, 2005) is presumably based on past experiences of offers being accepted or rejected and the emotions that were directly experienced as a result. The anticipated fear and guilt observed by Nelissen et al. (2011) stemmed from variations of the UBG that gave rise to heightened concern for self (fear) or concern for others (guilt). In the current research we examined a related but different issue. The point made by Nelissen and colleagues is that higher offers in the UBG may be driven by fear (of having one’s offer rejected) or guilt (about the opponent’s outcomes). Our objective is to show that *within* the context of fairness (which presumably enhances concern for others) or unfairness (which presumably reduces concern for others), the emotion one anticipates experiencing will shape one’s offer level. If you anticipate feeling proud about acting unfairly, you will offer less than you would if you anticipated feeling regret about acting unfairly. In contrast, if you anticipate feeling proud about acting fairly, you will offer more than you would if you anticipated feeling regret about acting fairly. To study this we examined the influence of both positive and negative anticipated emotions relating to both fair and unfair offers.

In the first of the present studies we manipulated anticipated emotions by first asking participants to engage in an autobiographical recall task. Anticipated emotions about fair or unfair behavior were measured before participants made an offer in the UBG. In the second study we investigated the effect of reminding participants about specific anticipated emotions (pride or regret) on subsequent fairness behavior. We manipulated anticipated pride and regret by having participants report their anticipated pride, their anticipated regret, or no emotion, before making an offer in the UBG. Both studies were approved by the Ethics Committee of Cardiff University’s School of Psychology.

## Study 1

### Method

#### Participants and design

The study had a 2 (Behavior: fair vs. unfair) × 3 (Emotion: pride vs. regret vs. control) between-subjects design, and was administered online. Participants were 210 people (131 female, 77 male, 2 undisclosed; age range: 18–77 years, median: 35 years; nationality: 85.7% British) who were recruited through an online loyalty program. As compensation for their time, participants received loyalty points that can be used for online shopping.

#### Materials

To manipulate behavior and anticipated emotion we asked participants to recall an incident from their own lives in which they had acted either in a way that was fair or unfair to others, and felt either proud or regretful as a result. Depending on behavior condition, we specifically asked participants to think back to a time when they behaved fairly (fair condition) or unfairly (unfair condition). Depending on emotion condition, we specifically asked participants to think back to a time when they felt proud (pride condition) or regretful (regret condition), “because you voluntarily gave up something that otherwise could have been yours” (fair condition) or “because you gained something for yourself that otherwise would not have been yours” (unfair condition). In the control condition participants also recalled an event in which they had acted fairly or unfairly, but no mention of emotions was made. As manipulation checks we asked participants to rate the extent to which they behaved fairly and unfairly in the recalled situation and the extent to which they had felt proud and regretful. The response scale for all measures ran from 1 (*not at all*) to 5 (*very much*).

Participants then played the UBG. This experimental game simulates a single-round negotiation; participants play for a resource that has monetary value. The game involves two roles, the “proposer” and the “responder.” The proposer divides the resource between the two players and this division is presented as an offer to the responder. The responder can accept or reject the offer. If the responder accepts the offer the resource is divided as proposed; if the responder rejects the offer neither player receives anything. In this study the resource for which participants played was £100, represented by 50 monetary units (MU) of £2 each. We explained to participants that at the end of the study we would randomly select two pairs of participants, and that we would divide the resource between the players in accordance with how they had played the game. Because we were interested in the number of MU that the proposer was willing to share with the responder as a measure of fair behavior, all participants were assigned the role of “proposer.”

Participants reported their anticipated emotions directly before playing the UBG. Because we were interested in anticipated emotions about fair and unfair behavior, we asked participants to report how they would feel if they were to divide the MU equally, or how they would feel if they were to keep most of the MU for themselves. Depending on behavior condition, we asked: “If you were to divide the MU equally between yourself and the responder (for example, if you would offer a 25–25 split), to what extent would you feel…” (fair condition), or “If you were to divide the MU in such a way that you keep most for yourself (for example, if you would offer a 45–5 split), to what extent would you feel…” (unfair condition). We asked participants to report their anticipated emotions on a scale from 1 (*not at all*) to 5 (*extremely*) for 10 different emotion terms: *pleased*, *proud*, *regretful*, *sorry*, *satisfied*, *relieved*, *embarrassed*, *foolish*, *guilty*, and *ashamed*.

#### Procedure

Participants first received general information about the study, confirmed that they were 18 years of age or older, and consented to participate in the study. Demographic information was collected, and participants completed a measure of Social Value Orientation (because this construct was not involved in our hypotheses, the results relating to this measure will not be reported). They then described the autobiographical event involving fair or unfair behavior and their experienced feelings of pride or regret (except in the control condition). In the next part of the study they learned about the rules of the UBG. Participants were led to believe that they were randomly assigned to their role; however, all participants were allocated to the role of “proposer.” Next, participants completed a set of comprehension checks that captured the most important aspects of the UBG (“What is your role in the game?”, “How many MU are there to divide?”, “How many MU will you receive if the offer is rejected?”) and received feedback on their answers to ensure that everyone was fully aware of the rules. Participants then reported their anticipated emotions, and made their offer in the UBG. After an open question about their thoughts and feelings concerning the game, participants indicated the minimum MU they would accept as an offer if they were a responder in the UBG. The £100 resource was paid to the randomly selected pairs of players in accordance with the responses they gave (e.g., if the participant selected as a proposer had offered a 30_proposer_:20_responder_ division of MU and the participant selected as a responder had indicated that he/she would accept a minimum offer of 35_proposer_:15_responder_ MU, then the £100 would be divided £60_proposer_:£40_responder_). Then participants completed manipulation checks and a second measure of Social Value Orientation. Finally, participants were thanked and debriefed.

### Results

#### Participants and data treatment

An independent judge, blind to condition, read the autobiographical reports of participants, and coded whether the stories made reference to fair or unfair behavior. Participants who did not provide an answer, could not think of a situation, or gave an unintelligible answer were excluded from analyses. One hundred fifty-two participants remained in the analyses. Three anticipated emotion items were combined into a single pride scale (*pleased*, *proud*, and *satisfied*; α = 0.90), and two items were combined into a single regret scale (*regretful* and *sorry*; α = 0.93).

#### Manipulation checks

We tested the effects of conditions on the manipulation checks with 2 (Behavior: fair, unfair) × 3 (Emotion: pride, regret, control) ANOVAs. The manipulation check for fair behavior revealed the expected main effect of the behavior manipulation, *F*(1, 146) = 42.29, *p* < 0.001, η^2^ = 0.23. In the fair condition, participants reported having behaved more fairly (*M *= 4.32, SD* *= 1.15) than in the unfair condition (*M *= 2.91, SD* *= 1.48). No other effects were significant. The reverse pattern was found for unfair behavior. As expected, participants reported behaving more unfairly in the unfair condition (*M *= 3.03, SD* *= 1.49) than in the fair condition (*M *= 1.61, SD* *= 1.11), *F*(1, 145) = 43.39, *p* < 0.001, η^2^ = 0.23, and no other effects were found.

The manipulation check for pride revealed the expected main effect of emotion condition, *F*(2, 146) = 12.14, *p* < 0.001, η^2^ = 0.14. Participants felt more proud in the recalled situation in the pride condition (*M *= 3.71, SD* *= 1.32) than in the regret (*M *= 2.37, SD* *= 1.51) or control conditions (*M *= 3.12, SD* *= 1.51). There also was a significant main effect of the behavior manipulation, *F*(1, 146) = 27.35, *p* < 0.001, η^2^ = 0.16, showing that participants felt more pride in the fair autobiographical stories (*M *= 3.57, SD* *= 1.36), than in the unfair stories (*M *= 2.40, SD* *= 1.52). The interaction was not significant. The manipulation check for regret revealed a similar pattern, but in the reverse direction. As expected, participants felt more regret in the recalled situation in the regret condition (*M *= 3.27, SD* *= 1.58) than in the pride (*M *= 2.63, SD* *= 1.52) or control conditions (*M *= 2.47, SD* *= 1.56), *F*(2, 146) = 3.36, *p* = 0.038, η^2^ = 0.04. Again, the main effect of behavior was significant, *F*(1, 146) = 13.42, *p* < 0.001, η^2^ = 0.08. Participants felt more regret in the unfair stories (*M *= 3.34, SD* *= 1.48) than in the fair stories (*M *= 2.39, SD* *= 1.54). The interaction was not significant.

#### Dependent variables

##### Anticipated emotions

We investigated the effect of behavior and emotion on anticipated pride and regret in two separate 2 × 3 ANOVAs. For anticipated pride the predicted main effect of emotion condition was not significant, *F* < 1, ns. Participants in the pride condition (*M *= 3.54, SD* *= 1.16) did not anticipate more pride than participants in the regret (*M *= 3.33, SD* *= 1.35) or control conditions (*M *= 3.59, SD* *= 1.17). However, there was a significant main effect of behavior, *F*(1, 144) = 70.39, *p* < 0.001, η^2^ = 0.33. Participants anticipated more pride in the fair (*M *= 4.08, SD* *= 0.94) than in the unfair condition (*M *= 2.66, SD* *= 1.10). The interaction was not significant. For regret, too, the predicted main effect of emotion condition was not significant, *F* < 1, ns. Participants in the regret condition (*M *= 2.41, SD* *= 1.49) did not anticipate more regret than participants in the pride (*M *= 2.19, SD* *= 1.15) or control conditions (*M *= 2.10, SD* *= 1.22). However, there was again a significant main effect of behavior condition, *F*(1, 140) = 145.49, *p* < 0.001, η^2^ = 0.51. Participants anticipated more regret in the unfair (*M *= 3.29, SD* *= 1.10) than in the fair condition (*M *= 1.43, SD* *= 0.74). The interaction was not significant.

##### Offer level

The number of MU allocated to the responder ranged between 5 and 30, with a median of 25, and a mean of 24.26. We investigated the effect of behavior and emotion on offer level in a 2 × 3 ANOVA. There were no significant effects (all *F*s ≤ 1.00).

**Figure 1:**
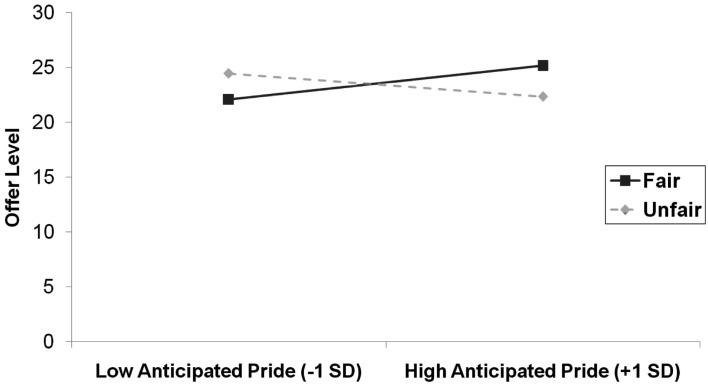
**Offer level (number of MU allocated to responder) as a function of anticipated pride and behavior condition in Study 1**.

We then investigated the combined effects of behavior condition, emotion condition, and self-reported anticipated emotion on offer level using multiple regression. We regressed offer level on behavior condition, emotion condition, self-reported anticipated emotion, and their interactions in two separate analyses: one with the measure of anticipated pride and its interaction terms, the other with the measure of anticipated regret and its interaction terms. We entered the main effects for the predictors in step 1 (*R*^2^ = 0.01, ns), the two-way interactions between these terms in step 2 (Δ*R*^2^ = 0.20, *p* < 0.001), and the three-way interaction term in step 3 (Δ*R*^2^ = 0.004, ns). For the regression involving anticipated pride there was a significant two-way interaction between behavior and self-reported anticipated emotion, β = 0.33, SE = 0.06, *p* < 0.001. This interaction is depicted in Figure 1. The simple slope of anticipated pride was significantly positive in the fair condition, β = 0.39, SE = 0.09, *p* < 0.001, while the simple slope of anticipated pride was significantly negative in the unfair condition, β = −0.27, SE = 0.09, *p* = 0.003. There were no other significant two-way interactions involving anticipated pride, and the three-way interaction between behavior condition, emotion condition, and anticipated pride was not significant.

**Figure 2:**
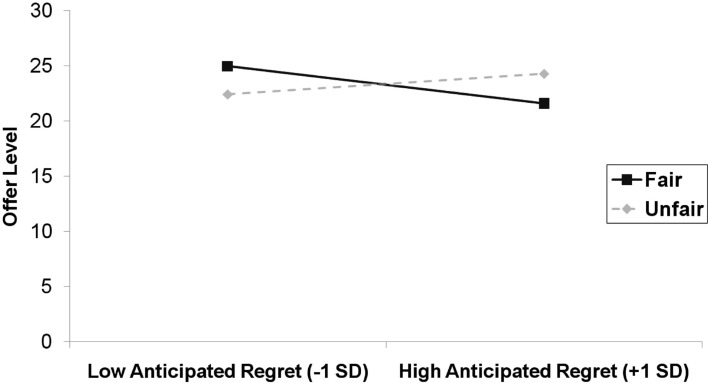
**Offer level (number of MU allocated to responder) as a function of anticipated regret and behavior condition in Study 1**.

For the regression that involved anticipated regret we again entered the main effects for the predictors in step 1 (*R*^2^ = 0.004, ns), the two-way interactions between these terms in step 2 (Δ*R*^2^ = 0.16, *p* < 0.001), and the three-way interaction term in step 3 (Δ*R*^2^ = 0.003, ns). Here, there was a significant two-way interaction between behavior and self-reported anticipated emotion in the opposite direction, β = −0.38, SE = 0.09, *p* < 0.001. This interaction is depicted in Figure 2. The simple slope of anticipated regret was significantly negative in the fair condition, β = −0.43, SE = 0.13, *p *= 0.001, while the simple slope of anticipated regret was significantly positive in the unfair condition, β = 0.24, SE = 0.10, *p* = 0.017. There were no other significant interactions that involved anticipated regret, and the three-way interaction between behavior condition, emotion condition, and anticipated regret was not significant.

#### Discussion

Despite the fact that the manipulation check data showed that participants recalled autobiographical events in accordance with the experimental instructions, the autobiographical recall task did not directly influence anticipated emotions and the manipulation had no direct effect on offer level. However, and in line with our predictions, the more that participants anticipated feeling proud about acting fairly, the more fairly they distributed the resources, whereas the more that they anticipated feeling proud about acting unfairly, the less fairly they distributed the resources. With regret the same pattern was found, but in reverse.

In better understanding why the autobiographical recall task did not influence anticipated emotions, it may be useful to consider the distinction between “exogenous” and “endogenous” emotion, as drawn by de Hooge et al. (2008), who argue that “Influences of emotions are denoted as endogenous when they concern behaviors in situations that are related to the emotion-causing event” (p. 935). In the present context, it could be argued that the autobiographically recalled event and its accompanying emotion were exogenous to the ultimatum game that participants played. Generalizing from the finding that exogenous shame is less likely than endogenous shame to influence prosocial behavior (de Hooge et al., 2008), it could be reasoned that the exogenous nature of the autobiographical recall procedure used here might have been responsible for the lack of influence on anticipated emotion in the UBG.

Another possible explanation for the fact that the manipulation was not successful in changing levels of anticipated emotion is that we measured anticipated emotions with a range of items and this may have served to “undo” the effect of the emotion manipulation. The results of the anticipated emotion measure suggested that it was easier to arouse pride in the fair than in the unfair condition, and regret in the unfair condition than in the fair condition. By asking participants to reflect on their anticipated emotions (and measuring both pride and regret), we may have led participants to revert to the “default” of anticipating pride in the fair condition, and regret in the unfair condition.

In Study 2 we therefore used a different manipulation of anticipated emotion: we measured only anticipated pride, or anticipated regret, or no emotion (as appropriate). In this way we ensured that the manipulation of anticipated emotion was endogenous to the experimental task participants had to complete. At the same time, by restricting the number of anticipated emotion items we ensured that there would be less interference from other emotion terms. We predicted that measuring pride in the fair condition would increase offer level, whereas measuring regret in the fair condition would decrease offer level. The reverse pattern of results was predicted in the unfair condition.

## Study 2

### Method

#### Participants and design

The study had a 2 (Behavior: fair vs. unfair) × 3 (Emotion: pride vs. regret vs. control) between-subjects design. Participants were 132 students of a British university (124 female, 7 male, 1 undisclosed; age range: 18–33 years, median: 19 years). Participants received course credit (as partial course requirement) in exchange for their time. The study was administered online.

#### Materials

We again used the UBG and the number of MU that proposers were willing to share as a measure of fair behavior. The resource (£1) was represented by 50 MU with a value of 2 pence each. Again, in a seemingly random assignment to the roles of “proposer” and “responder,” all participants were actually assigned the role of proposer. Because we did not collect information about the minimum offer that participants would accept (as we had done in Study 1) all participants received the maximum possible winnings (£1) at the end of the study in addition to their course credit.

Before participants divided the resource, they indicated their anticipated emotions. Depending on behavior condition we asked them to consider the following: “If you were to divide the MU equally between yourself and the responder (for example, if you would offer a 25–25 split), to what extent would you feel…” (fair condition), or “If you were to divide the MU in such a way that you keep most for yourself (for example, if you would offer a 45–5 split), to what extent would you feel…” (unfair condition). Depending on emotion condition, we asked participants to report either their anticipated pride (*pleased* and *proud*) or their anticipated regret (*regretful* and *sorry*) on a scale from 1 (*not at all*) to 5 (*extremely*). In the control conditions no anticipated emotion measure was administered. Instead, in the fair control condition participants were asked to consider dividing the MU equally, and in the unfair control condition to consider dividing the MU in such a way that they would keep most for themselves.

#### Procedure

After sign-up, participants received a link to the study website. On entering the website, participants received general information about the study and provided consent for participation. We recorded demographic information, and explained the UBG. We told them that their offer would be communicated to another participant by email, and participants provided their contact details for this purpose. All participants then learned that they were randomly allocated to the role of proposer. We checked for participants’ comprehension of the UBG using the same checks as in Study 1. Then participants reported their anticipated emotions, before making their offer in the UBG. Some additional measures were taken (e.g., an open question about their thoughts and feelings about the game, and Social Value Orientation), but because these measures are unrelated to the present hypotheses they will not be discussed further. Finally, participants were debriefed, provided with payment information and thanked.

### Results

#### Participants and data treatment

We excluded participants who shared all of their tokens (three participants) or kept everything for themselves (two participants) because such behavior likely reflects insufficient understanding of the game or lack of motivation to take the game seriously. For the remaining participants, offers ranged between 1 and 30 MU, with a median of 25, and a mean of 22.63. Anticipated emotion items were combined to create an anticipated pride scale (α = 0.70), and an anticipated regret scale (α = 0.88).

#### Dependent variables

##### Anticipated emotions

We investigated the effect of behavior condition and emotion condition on anticipated emotion with a 2 (Behavior: fair, unfair) × 2 (Emotion: pride, regret) ANOVA (because we did not collect anticipated emotion data in the control conditions, these were not included in the ANOVA). There was a significant main effect of behavior, *F*(1, 76) = 9.71, *p* = 0.003, η^2^ = 0.11, and a significant main effect of emotion, *F*(1, 76) = 24.60, *p* < 0.001, η^2^ = 0.25, but these main effects were qualified by a significant behavior by emotion interaction, *F*(1, 76) = 56.23, *p* < 0.001, η^2^ = 0.43. Simple main effects revealed that participants anticipated more pride in the fair (*M *= 3.67, SD* *= 0.58) than in the unfair condition (*M *= 2.84, SD* *= 1.20), *F*(1, 76) = 8.77, *p* = 0.004, whereas participants anticipated more regret in the unfair (*M *= 3.32, SD* *= 0.86) than in the fair condition (*M *= 1.31, SD* *= 0.52), *F*(1, 76) = 39.50, *p* < 0.001.

##### Offer level

**Figure 3:**
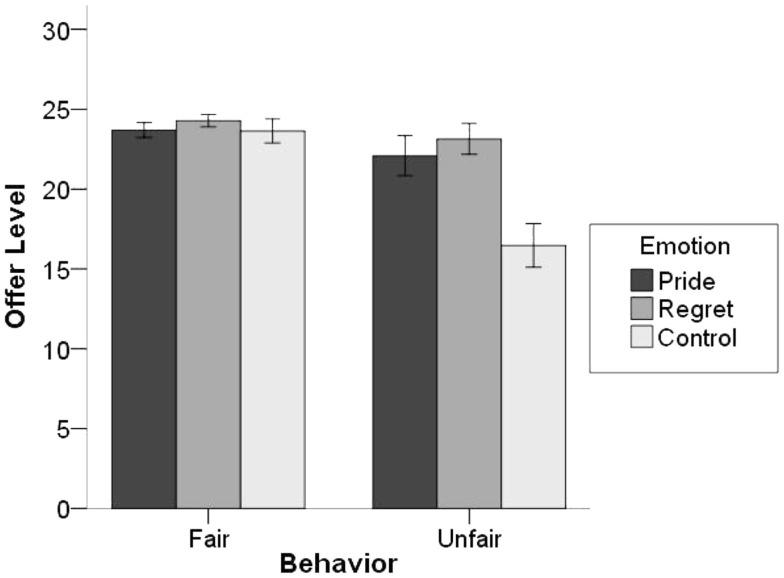
**Offer level (number of MU allocated to responder) as a function of behavior and emotion condition in Study 2**. Error bars represent ± 1 SE.

We investigated the effect of behavior and emotion on offer level in a 2 × 3 ANOVA. There was a significant main effect of behavior, *F*(1, 121) = 19.36, *p* < 0.001, η^2^ = 0.14, and a significant main effect of emotion, *F*(2, 121) = 8.52, *p* < 0.001, η^2^ = 0.12. These effects were qualified by a significant two-way interaction, *F*(2, 121) = 6.53, *p* = 0.002, η^2^ = 0.10 (see Figure 3). Simple main effects revealed that there was no effect of emotion in the fair condition (*F *< 1, ns; *M*_pride_ = 23.70, SD_pride_ = 2.25; *M*_regret_ = 24.29, SD_regret_ = 1.79; *M*_control_ = 23.64, SD_control_ = 3.51), but that there was a significant effect of emotion in the unfair condition, *F*(2, 121) = 11.69, *p* < 0.001. Follow-up analyses revealed that in the unfair condition participants offered fewer MU in the control condition (*M*_control_ = 16.47, SD_control_ = 5.96) than in either the pride (*M*_pride_ = 22.09, SD_pride_ = 5.86; *p* < 0.001), or the regret conditions (*M*_regret_ = 23.15, SD_regret_ = 4.36; *p* < 0.001).

**Figure 4:**
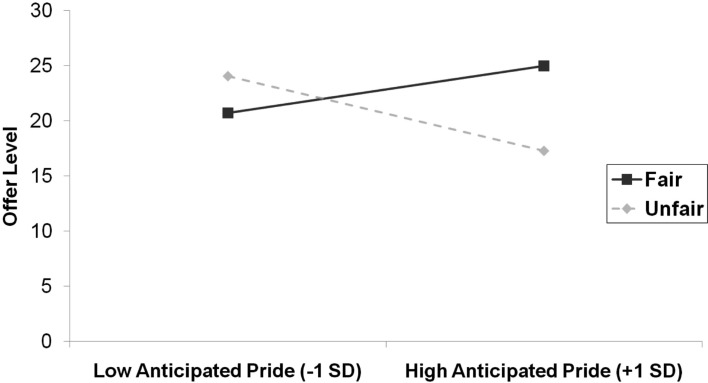
**Offer level (number of MU allocated to responder) as a function of anticipated pride and behavior condition in Study 2**.

**Figure 5:**
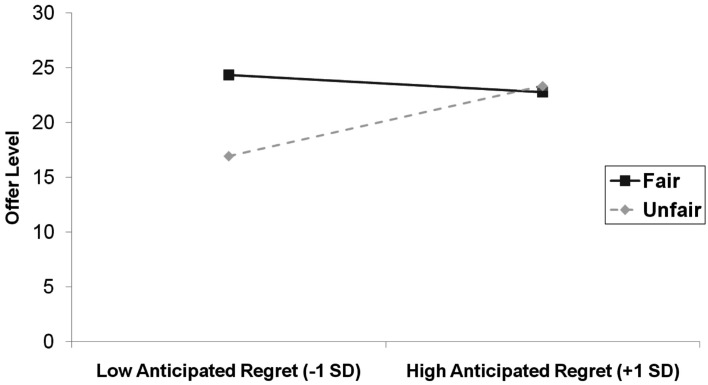
**Offer level (number of MU allocated to responder) as a function of anticipated regret and behavior condition in Study 2**.

We again investigated the combined effect of behavior, emotion, and the anticipated emotion measure on offer level using multiple regression. We entered the main effects for the behavior and emotion conditions and anticipated emotions in step 1 (*R*^2^ = 0.06, *p *= 0.17), the two-way interactions between these terms in step 2 (Δ*R*^2^ = 0.20, *p* < 0.001), and the three-way interaction term in step 3 (Δ*R*^2^ = 0.12, *p *< 0.001). This revealed a significant three-way interaction, β = 0.67, SE = 0.18, *p* < 0.001. To decompose this interaction we regressed offer level on behavior, anticipated emotion, and its interaction separately for each emotion condition. We entered the terms for the main effects in step 1, and their interaction term in step 2. The results replicated the pattern observed in Study 1. In the pride condition (step 1: *R*^2^ = 0.26, *p *= 0.002, step 2: Δ*R*^2^ = 0.20, *p* < 0.001) there was a significant two-way interaction between behavior and anticipated emotion, β = 0.70, SE = 0.18, *p* < 0.001. This interaction is depicted in Figure 4. Simple slopes revealed that while there was a trend for a positive association between anticipated pride and offer level in the fair condition, β = 0.54, SE = 0.32, *p* = 0.10, there was a negative association between anticipated pride and offer level in the unfair condition, β = −0.86, SE = 0.16, *p* < 0.001. For regret (step 1: *R*^2^ = 0.15, *p *= 0.076, step 2: Δ*R*^2^ = 0.08, *p* = 0.082) there was a marginally significant two-way interaction between behavior and anticipated emotion in the reverse direction, β = −0.51, SE = 0.28, *p* = 0.082. This interaction is depicted in Figure 5. Simple slopes revealed that there was no association between anticipated regret and offer level in the fair condition, β = −0.20, SE = 0.48, ns, whereas there was a significant positive association between anticipated regret and offer level in the unfair condition, β = 0.82, SE = 0.30, *p* = 0.010.

#### Discussion

As in Study 1, there was a clear relation between anticipated emotions and the subsequent offer, and the direction of this relation depended on the fair/unfair context: in the fair condition, the more participants anticipated to feel pride, the more MU they tended to allocate to the responder; whereas in the unfair condition, the more participants anticipated to feel pride, the less MU they tended to allocate to the responder. The reverse was found for anticipated regret, although in the fair condition the negative relation between anticipated regret and number of MU allocated to the responder did not reach significance.

Overall, participants anticipated more pride in the fair condition than in the unfair condition, and anticipated more regret in the unfair condition than the fair condition. In particular, there was very little anticipated regret in the fair condition. The latter may help to account for the non-significance of the simple slope of regret in the fair condition.

Asking people about either anticipated pride or anticipated regret increased the offer level in the unfair condition. Although this pattern of results for the direct effects of the behavior condition and the emotion condition on offer level differed from the one we had originally predicted, it can nevertheless be seen as consistent with our general theorizing. At first glance it may seem surprising that both emotion conditions led to higher offers in the unfair condition. However, when we take into account the direct effects of behavior condition on anticipated emotions, we can see that participants reported low levels of anticipated pride and high levels of anticipated regret in the unfair condition. Because low pride and high regret are related to higher offers in the unfair condition, it would appear that both emotion conditions (pride and regret) made participants think about how they would feel after acting unfairly, and that this increased offer levels in both cases. Although this interpretation is *post hoc* and therefore remains tentative, it is consistent with the fact that when participants were asked to consider making an unfair offer but were not asked to report their anticipated emotions, the result was a significantly lower average offer.

## General Discussion

In two studies we found that participants’ decisions about how to allocate resources between self and other are associated with the emotions that are anticipated as a result of their decision. The more that participants anticipated feeling proud about acting fairly, the higher were the offers they made to anonymous others; the more that participants anticipated feeling regret about acting fairly, the lower were the offers they made to anonymous others. Likewise, the more that participants anticipated feeling proud about acting unfairly, the lower were the offers they made to anonymous others; and the more that participants anticipated feeling regret about acting unfairly, the higher were the offers they made to anonymous others. These findings are consistent with the argument that decision makers take the emotional consequences of their decisions into account when making decisions (Mellers et al., 1999; Loewenstein and Lerner, 2003), and with the broader argument that anticipated emotions shape behavior (Baumeister et al., 2007).

Interestingly, the fact that participants who anticipated feeling proud about fair behavior or regret about unfair behavior were willing to part with some of their potential material winnings demonstrates that future emotions can be as important to participants as potential monetary rewards. This shows that people not only strive to maximize their gains, but also strive to feel good (or to not feel bad). In this sense positive emotions (or absence of negative emotions) can compensate for material loss or be an additional incentive for material gains. This means that decisions that people make when distributing resources between themselves and another person are better understood when anticipated emotions are taken into account.

It is noteworthy that overall participants more readily anticipated pride in relation to the prospect of behaving fairly and regret in relation to the prospect of behaving unfairly. This reflects the fact that the “default” decision in the UBG is to distribute the resources equally between proposer and responder. The modal proposed division of resources is 50:50 and very unfair offers are rare (Güth et al., 1982; Messick, 1993; Camerer, 2003). Despite some cross-cultural variability, this basic pattern has even been replicated in small-scale societies (Henrich et al., 2005), and has been interpreted as reflecting a social preference for inequity aversion (Fehr and Schmidt, 1999; Bolton and Ockenfels, 2000).

In discussing the results of Study 1 we argued that the fact that we measured a range of anticipated emotions (tapping both pride and regret) may have led participants to think more generally about the emotions that they expected to experience as a function of their resource allocation decisions, thereby undoing the influence of the recalled emotion. In Study 2 we therefore asked only about one anticipated emotion construct (pride or regret, plus a no emotion control condition). We found that in the unfair condition – where participants were asked to contemplate a 45:5 split in favor of them – asking about *either* pride *or* regret led to increased offers relative to the control condition. Thus, rendering future emotions salient led to fairer decisions irrespective of the specific emotion on which participants focused. This suggests that interventions aimed at making people think about how they will feel if they behave one way or the other may increase the probability of decisions that conform to the socially normative behavior in the respective situation. We argue that this is because – once they are led to think about it – most people will feel better about engaging in, rather than acting contrary to, the behavior that they consider to be socially normative. It would appear that the social preference for inequity aversion is sufficiently strong and widespread in society that participants anticipate, on average, less pride and more regret in relation to unfair allocations than in relation to fair allocations, with the result that offer levels were higher in both conditions. Whether alternative manipulations could not only change the saliency of future emotional states, but also induce specific anticipated emotions that exert discrete effects on behavior is an important challenge for future research in this area.

But even if our manipulation simply affected the salience of anticipated emotions, we believe that the practical implications of our findings are potentially substantial. Note that the manipulation we used was short and easy to administer. People were simply asked to consider what they would feel if they were to behave one way or the other. The result of this (irrespective of which emotion they focused on) was that they behaved in a fairer manner than they did when only considering the outcome of their choices. Whether similar interventions reminding people of the possible emotional consequences of their actions and inactions could have socially beneficial effects when printed on tax return forms, library books, office pens, or communal kitchen sinks is worth future research attention.

The present research illustrates one important yet relatively neglected way in which people can regulate their emotions in interpersonal settings: in order to regulate their feelings people modify the situation (Gross and Thompson, 2007). People anticipate how their actions will affect the self and others, and how they themselves are likely to feel as a result. This then influences the decisions that people make. The particular forecast of how they are likely to feel may be informed by past experiences in similar situations. Importantly, however, the absolute accuracy of this forecast of their feelings is unimportant, as long as it is relatively accurate in the sense that it indexes whether a given outcome is more likely to give rise to feelings of (for example) regret than of pride.

In conclusion, when making resource allocation decisions people take into account how they would feel if they were to do this in ways that vary with respect to fairness, and then make allocations that are informed by these anticipated emotions. In this way, people regulate their own emotions in social situations, giving themselves the opportunity to experience positive emotions such as pride and avoiding the experience of negative emotions such as regret. Interestingly, these anticipated emotions are enough of an incentive for people to sacrifice potential monetary gains. The pride people anticipate about acting fairly leads people to act fairly.

## Conflict of Interest Statement

The authors declare that the research was conducted in the absence of any commercial or financial relationships that could be construed as a potential conflict of interest.
